# Supplementary material for: Combined polygenic risk scores of different psychiatric traits predict general and specific psychopathology in childhood
Source: J Child Psychol Psychiatry. 2021 Aug 13;63(6):636–45. doi: 10.1111/jcpp.13501 (PMC9291767; doi:10.1111/jcpp.13501)
Supplement: Supplementary file 1 — Figure S1. Polygenic risk scores selection flowchart. [file JCPP-63-636-s001.pdf]

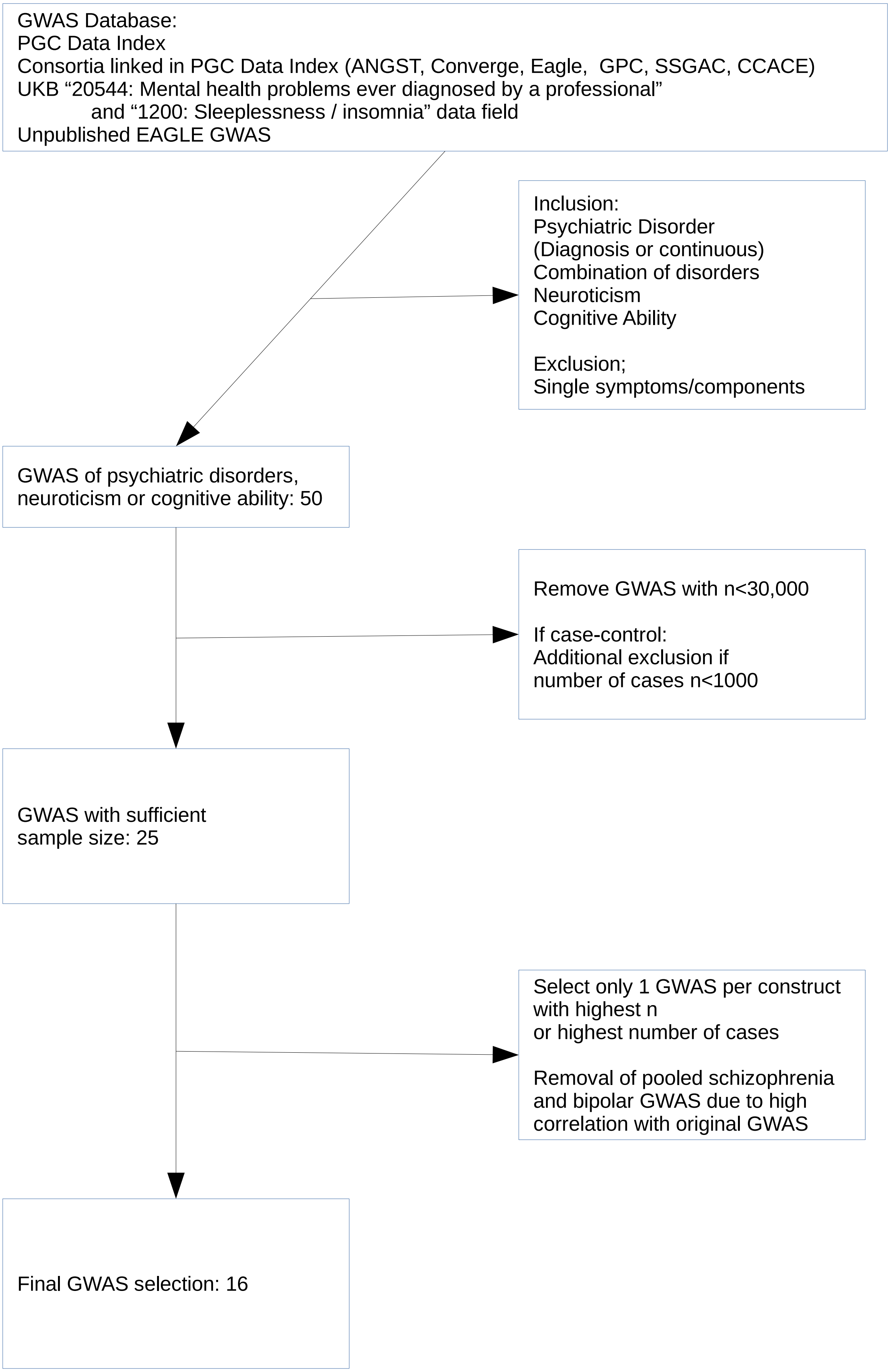
GWAS Database:
PGC Data Index
Consortia linked in PGC Data Index (ANGST, Converge, Eagle, GPC, SSGAC, CCACE)
UKB “20544: Mental health problems ever diagnosed by a professional” and “1200: Sleeplessness / insomnia” data field
Unpublished EAGLE GWAS
Inclusion:
Psychiatric Disorder
(Diagnosis or continuous)
Combination of disorders
Neuroticism
Cognitive Ability
Exclusion;
Single symptoms/components
GWAS of psychiatric disorders, neuroticism or cognitive ability: 50
Remove GWAS with n<30,000
If case-control:
Additional exclusion if number of cases n<1000
GWAS with sufficient sample size: 25
Select only 1 GWAS per construct with highest n or highest number of cases
Removal of pooled schizophrenia and bipolar GWAS due to high correlation with original GWAS
Final GWAS selection: 16
